# Quality and readability of web-based Arabic health information on COVID-19: an infodemiological study

**DOI:** 10.1186/s12889-021-10218-9

**Published:** 2021-01-18

**Authors:** Esam Halboub, Mohammed Sultan Al-Ak’hali, Hesham M. Al-Mekhlafi, Mohammed Nasser Alhajj

**Affiliations:** 1 Department of Maxillofacial Surgery and Diagnostic Sciences, College of Dentistry, Jazan University, Jazan, Kingdom of Saudi Arabia; 2 Department of Oral Medicine, Oral Pathology and Oral Radiology, Faculty of Dentistry, Sana’a University, Sana’a, Yemen; 3 Department of Preventive Dental Sciences, College of Dentistry, Jazan University, Jazan, Kingdom of Saudi Arabia; 4 Department of Periodontology, Faculty of Dentistry, Sana’a University, Sana’a, Yemen; 5 Medical Research Center, Jazan University, Jazan, Kingdom of Saudi Arabia; 6 Department of Parasitology, Faculty of Medicine and Health Sciences, Sana’a University, Sana’a, Yemen; 7 Department of Parasitology, Faculty of Medicine, University of Malaya, 50603 Kuala Lumpur, Malaysia; 8 Department of Prosthodontics, Faculty of Dentistry, Thamar University, Dhamar, Yemen

**Keywords:** COVID-19, Infodemiology, Health information, Misinformation, Public health, Quality

## Abstract

**Background:**

This study sought to assess the quality and readability of web-based Arabic health information on COVID-19.

**Methods:**

Three search engines were searched on 13 April 2020 for specific Arabic terms on COVID-19. The first 100 consecutive websites from each engine were analyzed for eligibility, which resulted in a sample of 36 websites. These websites were subjected to quality assessments using the Journal of the American Medical Association (JAMA) benchmarks tool, the DISCERN tool, and Health on the Net Foundation Code of Conduct (HONcode) certification. The readability of the websites was assessed using an online readability calculator.

**Results:**

Among the 36 eligible websites, only one (2.7%) was HONcode certified. No website attained a high score based on the criteria of the DISCERN tool; the mean score of all websites was 31.5 ± 12.55. As regards the JAMA benchmarks results, a mean score of 2.08 ± 1.05 was achieved by the websites; however, only four (11.1%) met all the JAMA criteria. The average grade levels for readability were 7.2 ± 7.5, 3.3 ± 0.6 and 93.5 ± 19.4 for the Flesch Kincaid Grade Level, Simple Measure of Gobbledygook, and Flesch Reading Ease scales, respectively.

**Conclusion:**

Almost all of the most easily accessible web-based Arabic health information on COVID-19 does not meet recognized quality standards regardless of the level of readability and ability to be understood by the general population of Arabic speakers.

**Supplementary Information:**

The online version contains supplementary material available at 10.1186/s12889-021-10218-9.

## Background

Coronavirus disease 2019 (COVID-19) has been a terrifying disease since it first appeared in December 2019 in Wuhan, China. The causative pathogen was identified as severe acute respiratory syndrome coronavirus 2 (SARS-CoV-2) [1, 2]. In March 2020, the disease was classified as a pandemic by the World Health Organization (WHO), and over the following months it spreads exponentially affecting almost every country in the world, prompting the imposition of curfews and lockdowns aimed at limiting the community spread of the disease [3, 4]. During such crisis, people crave news.

Under the current circumstances, people are eager to find out everything they can about COVID-19: the numbers of new and critical cases, and related deaths; the performance of health systems; the preventive measures announced by the relevant authorities; the availability of therapeutic remedies and vaccines; the innovations and new policies being implemented to fight the disease and so on [5]. Given that access to and use of the World Wide Web (web, internet or net) is now widespread, people have turned to this resource as it holds lots of information. However, not all of this information can be trusted equally as the sources range from personal or group opinions to scientific articles in peer-reviewed journals [6–8].

On the face of it, the web can be described as a giant step forward for humankind in terms of its capacity for information gathering and dissemination. Indeed, it could be said that the phrase “Just Google it” has become the first response when faced with a request for unknown information or asked to provide the answer to all types of questions. Theoretically, the web seems to be a very good tool for the public to use to obtain additional medical information that they do not know about their conditions or, in the current context about the COVID-19 pandemic [9, 10]. However, thousands or even millions of websites appear with every single-word search, but only a few of them are relevant to what the user is looking for, and not many of those are necessarily of suitable quality in terms of the information they contain. Regrettably, the public does not know which websites are trustworthy and which are not, and despite the fact that there are certified medical/health websites, they are very few in number [11]. Moreover, access to scientific articles, which are trustful, is limited and requires the payment of a subscription in most instances, and they use complex scientific terms and concepts that are generally difficult for the public to understand. The net result of the above constraints might be that people obtain inaccurate or misleading information, which might lead to the subsequent adoption of unhealthy behaviors, such as using unapproved drugs or harmful herbs, and applying inappropriate preventive measures. The problem of quality of the web-based health information is not language-exclusive, although its impact might be less obvious among English-speaking people due to the fact that most of the scientific output is published in English, and very few are translated after a while into other languages.

In the Arabic world, very few people speak/read English, and no certified Arabic medical websites are available, except for those of international organizations that translate their content into different languages [12–16]. At a time when the Arabic world as elsewhere is continuing to combat COVID-19, many Arabic medical, educational, social, news, and even sports websites are publishing materials regarding the disease. Therefore, the present study sought to assess the quality and readability of such online Arabic health information on COVID-19 in order to determine whether this information is of benefit to the public.

## Methods

The present study adopted an infodemiological approach in which selected search engines were searched for specific Arabic terms on COVID-19 and the selected websites were then subjected to quality and readability assessments using well-established tools.

### Search strategy

The search for websites was conducted on 13 April 2020. The cookies information was erased from the browser prior to starting the search. To prevent any biases arising from preceding searches, browsing was done using Incognito (InPrivate) mode. Using Google Chrome version 81.0.4044, the three following engines were searched: Google (http://www.google.com), Yahoo! (http://www.yahoo.com), and Bing (http://www.bing.com). The most widely used Arabic translations of the following words were used as search keywords: Coronavirus, Corona, and COVID-19. The following combination was used in the Google search engine: “Coronavirus-فيروس كورونا” OR “Corona-كورونا” OR “COVID-19-كوفيد-19”. When agreement on the search strategy had been reached, each engine was searched by one of the authors.

**Fig. 1:**
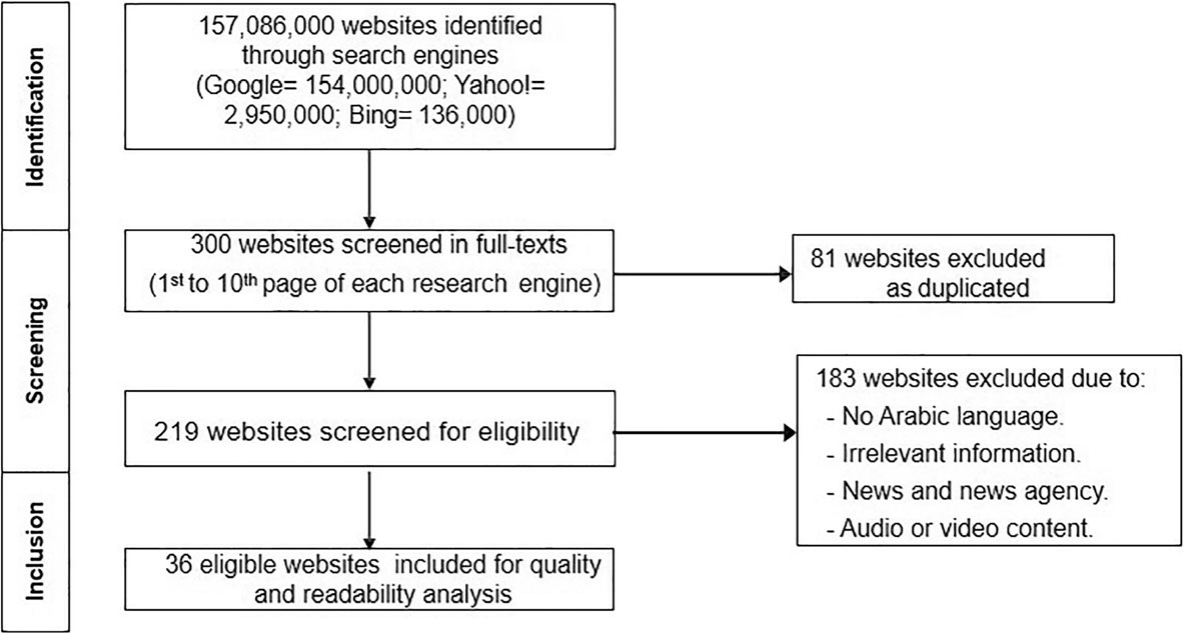
Different stages of the search strategy that was followed by the study

This initial search was limited to the first 100 consecutive websites (the first 10 consecutive pages) obtained from each engine. These websites from the three engines were then checked for duplicates, which, when found, were removed. The websites that presented health information on COVID-19 in the Arabic language were selected for subsequent evaluation. The following exclusion criteria were applied to identify relevant websites: 1) language other than Arabic; 2) information on COVID-19 just by hints, or exclusively audio or visual-based; 3) complete scientific articles or textbook; 4) banner advertisements or sponsored links and discussion forums; 5) blocked sites, or sites with denied direct access (requiring ID and password); 6) no information about COVID-19; and 7) News and news agencies, and social media. The remaining websites were included and assessed for quality and readability, as indicated below. Figure 1 depicts the different stages of the search strategy that was followed.

### Quality assessment

The quality of the included websites was evaluated using the DISCERN [17], the Journal of the American Medical Association (JAMA) benchmarks [18], and the Health on the Net Foundation Code of Conduct (HONcode) [19] assessment tools.

The DISCERN tool is a questionnaire that contains 16 questions. It is structured into three sections: Questions 1–8 address whether the website can be trusted as a source of data about a selected therapy; questions 9–15 are about therapy options, and question 16 measures the overall quality score at the end of the evaluation. Each question is scored from 1 to 5, where 1 indicates a poor website, and 5 indicates a good-quality website. The overall score ranges from 16 to 80: ≥ 65 is considered high quality; 33–64 is considered moderate quality; and 16–32 is considered low quality.

The JAMA benchmarks tool evaluates the following four criteria: 1) authorship (whether authors, their contributors, affiliations, and relevant credentials are displayed or not); 2) attribution (whether clear references and sources for the content are provided or not); 3) disclosure (whether ownership, sponsorship, advertising, underwriting, commercial funding or support sources and any potential conflicts of interest are displayed or not); and 4) currency (whether dates of initial posting and updating of the content are mentioned or not). For each fulfilled criterion, the website scores 1 point; otherwise, it scores 0 points. The score ranges from 0 to 4 points as follows: “0 = No item met”; “1 = One item met”; “2 = Two items met”; “3 = 3 items met”; and “4 = 4 items met.”

The HONcode is a certificate that a website can obtain by complying with a set of criteria. If those criteria are met, the website is granted permission to display a stamp (an HON award-like badge) on its pages. This certificate is valid for 1 year only.

The quality assessment using DISCERN and JAMA was conducted by two authors (EH and MSA). To minimize subjectivity, both authors assessed five websites together using these two tools, and they resolved any discrepancies by discussion. Moreover, later on, inter-examiner calibration was calculated for all the assessed websites. As regards the HONcode elements of the quality assessment, the HONcode software was downloaded and incorporated as an extension into Google Chrome. Then, with each search, a HONcode seal appeared on the websites that were certified. The currency of the HONcode seal on these websites was checked by referring to the main HONcode website.

### Readability assessment

The guidelines of the American Medical Association and the US Department of Health and Human Services were consulted for the readability component of the assessment. These guidelines recommend that patient reading material should be more accessible and understandable by the general public, and should not be higher than 5th or 6th grade reading level [20, 21]. The readability of the website materials was assessed using an online readability calculator tool developed by Online Utility (http://www.online-utility.org/english/readability_test_and_improve.jsp). Although this tool was primarily designed to analyze English language text, it can be used for other languages, as indicated on the Online Utility website. Moreover, before commencing the study, the authors tested the validity of this tool using Arabic texts. Three Arabic paragraphs of three different levels of difficulty (simple, medium, and difficult) were analyzed. The results revealed values that corresponded to the difficulty of the respective texts. The tool has also been validated by a previous study [22]. The website-based tool analyzes text using different common, well-known analysis tools (Gunning Fog Index (GFI), Coleman Liau Index (CLI), Flesch Kincaid grade level (FKGL), Automated Readability Index (ARI), Simple Measure of Gobbledygook (SMOG), and Flesch Reading Ease (FRE). The GFI, CLI, and ARI were not considered in the analyses because these indices use the number of letters to formulate the readability score. This formula is not applicable to Arabic text as, unlike an English word that is composed of separate letters, the Arabic word is composed of different components, such as morphemes, that are linked together. The acceptable readability level was set as ≥ 80.0 for the FRE and < 7 for the FKGL and SMOG [20, 21].

## Results

The web search revealed a total of 157,086,000 results. Out of the 300 websites that were selected (i.e. the first 100 listed by each of the three search engines, Google, Yahoo, and Bing), 81 websites were excluded as duplicates. The remaining 219 websites were analyzed for eligibility based on the seven aforementioned exclusion criteria. As a result, 183 websites were excluded. Thus, 36 eligible websites were included in the quality and readability analyses (see Additional file 1: Table S1).

With regard to HONcode certification, only two (5.5%) websites (who.int and mayoclinic.org) displayed the HONcode seal. However, a check of the HONcode website revealed that the certificate for the who.int website was expired (https://www.hon.ch/HONcode/Conduct.html? HONConduct982312). Hence only one website (2.7%) was HONcode certified. In respect of the results of applying the DISCERN tool to the websites, it was found that none of the websites achieved a high-quality score (≥ 65). However, 13 (36.1%) websites had a moderate score (33–64). The remaining 23 (63.9%) had a low score (16–32). The mean score for all websites was 31.5 ± 12.55.

As for the results of using the JAMA benchmarks tool, only four (11.1%) websites met all the JAMA criteria (i.e., scored 4 out of 4). Two (5.6%) websites scored 0 (did not fulfil any of JAMA criteria). The majority (41.7%) of the analyzed websites had a score of 2 (met two JAMA criteria). The mean score for all websites was 2.08 ± 1.05. As regards compliance with the specific benchmark criteria, while most of the websites did not display information on authorship and attribution, they did display information on disclosure and currency (Table 1).

**Table 1:** Quality assessment of the included websites (*n* = 36)

Criteria	Frequency	Percent
**HONcode**		
Certified	1	2.8
Not certified	35	97.2
**DISCERN**		
High (≥ 65)	0	0.0
Moderate (33–64)	13	36.1
Low (16–32)	23	63.9
**JAMA Benchmarks**		
No item met	2	5.6
One item met	8	22.2
Two Items met	15	41.7
Three items met	7	19.4
Four items met	4	11.1
**Authorship - JAMA**		
0 (Not met)	20	55.6
1 (Met)	16	44.4
**Attribution - JAMA**		
0 (Not met)	23	63.9
1 (Met)	13	36.1
**Disclosure - JAMA**		
0 (Not met)	12	33.3
1 (Met)	24	66.7
**Currency - JAMA**		
0 (Not met)	14	38.9
1 (Met)	22	61.1

In regard to the above DISCERN and JAMA results, the inter-examiner agreement according to the Kappa values were 0.88 and 0.95 for the DISCERN tool and the JAMA benchmarks tool, respectively.

In relation to the issue of readability, the mean grade level based on the FKGL was 7.2 ± 7.5. Most of the included websites (66.7%) had scores < 7, implying that the content was easy for the general public to understand. The unicef.org website had the most difficult content (FKGL = 46.72). When this website was excluded, the mean grade level dropped to a score of 6.0 ± 3.1. According to the SMOG score, the grade level needed to understand the text of the websites ranged from 3 to 5.3, with a mean grade level of 3.3. As for readability ease, the FRE mean score was 93.5 ± 19.4, indicating that the content of thee websites was easy for the general public to read. Again, the website unicef.org had the most complex text (FRE = − 9.21). When this website was excluded, the mean FRE score increased to 96.4 ± 8.2. More details are presented in Table 2.

**Table 2:** Readability assessment of the websites included in the present study (n = 36)

	Flesch Kincaid Grade level	Simple Measure of Gobbledygook (SMOG)	Flesch Reading Ease
Mean	7.2	3.3	93.5
SD	7.5	0.6	19.4
Minimum	2.8	3	−9.2
Maximum	46.7	5.3	105
< 7 score	66.7% (*n* = 24)	83.3% (*n* = 30)	NA
≥ 7 score	33.3% (*n* = 12)	16.7% (*n* = 6)	NA
≥ 80 score	NA	NA	94.4% (*n* = 34)
< 80 score	NA	NA	5.6% (*n* = 2)

*NA* Not Applicable

## Discussion

As COVID-19 is a novel and virulent disease, it has been trending news in all media and websites worldwide since its emergence, and Arab social media and websites have been no exception. Given the critical nature of the COVID-19 pandemic, the present study sought to assess the quality and readability of the health information on COVID-19 provided by Arabic websites. The most famous search engines in the Arabic world were searched and the first 100 websites from each engine were obtained, although users mostly do not go beyond the first 20 websites [23–26]. The small number of websites included in the analyses due to the exclusion of news, and news agencies and social media. During a pandemic, such as the COVID-19, these are the most frequent sources of information, at least from the users’ point of view [27–29]. However, these sources just broadcast and/or publish what they get from the responsible sources, along with information from their special (unknown) sources. Apart from the daily reports of new cases and deaths, the relevant information from the health point of view is that which is related to the ways in which the virus spreads, the signs and symptoms of the disease, the required preventive measures and guidelines, and the available treatment and vaccines. Unfortunately, such information is hard to find, or inappropriately, covered in the news and by news agencies and social media. Hence, these websites were excluded from the analyses [26, 29, 30].

As the present study is, to the best of our knowledge, the first to assess the quality and readability of web-based Arabic content on COVID-19, comparison with other findings in the available literature is limited. Moreover, the assessment of web-based Arabic medical information has been scarcely addressed and where such assessments do exist, they have focused on medical conditions other than COVID-19, such as oral cancer [16], breast cancer [15], epilepsy [14], autism [13], denture hygiene [22], and oral health [12]. Yet, strikingly, these studies unanimously agree on the poor quality of the web-based Arabic information about these diseases.

In the present study, only one website was found to be HONcode certified. Surprisingly, the HONcode certificate of the WHO website was invalid (expired). As a nonprofit and nongovernmental organization, HONcode aims at promoting transparent and reliable health information online and issues its certificates based on a minimum mechanism to provide good-quality, objective, and transparent medical information to internet users. The certified websites have the right to display the HONcode seal; this means that they agree to comply with the standards listed and are subjected to random audits on compliance [31].

With regard to quality of the websites according to the DISCERN tool, no single website achieved a high score. Most of the shortcomings of the included websites can be attributed to the second section of the DISCERN questionnaire (questions 9–15 on therapy options) as data about treatments, alternatives, side effects etc. of the proposed drugs were scarcely or improperly covered. To a lesser extent, the first section (question 1–8 on website trustworthiness) also contributed to the low-quality scores as: no or scarce data were available on the aims of presenting the website contents or on how these aims—when present—were achieved; the relevance of the topic; the source of information; the date of publication; the degree of bias or balance; and areas of uncertainty. The net result of these shortcomings was a lower score for the last question (on the overall quality of the content). The shortcomings relation to the second section might be ascribed to the fact that the disease is novel, and no confirmed treatments and alternatives being available at the time of the study. However, the low-quality score of the first section cannot be attributed to the same reason. Hence, the websites should be able to fulfill these criteria for any written content they publish.

According to the JAMA benchmarks tool, the mean score for quality was 2.08 ± 1.05. This is a poor score. Most of the shortcomings in the respect of the JAMA criteria arise from the included websites not providing information on the authorship and attribution of their content. On the other hand, most of the websites displayed information on the disclosure and currency of their content. It is strange that health topics are published on websites without mentioning the authors and references.

The quality assessment was not as expected. The analyses revealed that the information was lower than the quality standards required for health information, and hence it can be said that the information on these websites was not entirely reliable. Similar results for health information on COVID-19 were reported by Cuan-Baltazar et al. for English and Spanish websites, but it should be noted that news agencies and social media were included in the analysis [5]. As the disease is more serious in Europe and the USA, in terms of incident cases and associated deaths, it might be expected that the quality of the English and Spanish health information about COVID-19 would be higher than the Arabic ones. However, this does not seem to be the case.

The apparent widespread availability of poor-quality information is at best misleading, and at worst dangerous, especially in the context of the COVID-19 pandemic as this disease is so threatening to everyone, prompting them to believe in what they read, despite the poor quality of the information, and turn it into a practice that may eventually be harmful [6, 29]. The picture is dark and gets darker if the sites that have been excluded (such as news agencies and social media) were considered. Further, the scientific information about COVID-19 seems to be full of flaws owing to the fact that the disease is novel, so, as yet, no full picture of its etiopathogenesis, clinical manifestations, laboratory findings, preventive and treatment measures is to hand [10]. In these circumstances, Hernández-García et al. [32] argued that “It is necessary to urge and promote the use of the websites of official public health organizations when seeking information on COVID-19 preventive measures on the internet.”

With regard to readability, the analyses of the current study revealed that most of the websites contained simple text that can be read and understood by most of the general public. Hence, it is discouraging that most of the websites provide poor-quality health information, which because it is simple to read and understand, jeopardizes the readers. It is advantageous to have websites that provide information on topics in simple terms can be understood by most people, but it is also disastrous considering the poor quality of these content on these important topics.

In light of the above, the authorities must undertake initiatives that aim at monitoring, controlling, and enhancing web-based health information, with special emphasis on the current pandemic—COVID-19. In addition, the authorities must be given the powers to administer the necessary punitive measures against violating entities when they publish web-based health information that falls below the required quality. Website developers should provide the reader with more reliable, simple, and readable content. At the very least, they should include the source of the information, date of publication, name of author or writer, and short sentences that employ simple and clear terminology.

## Conclusions

Almost all of the most easily accessible web-based Arabic health information on COVID-19 does not meet standards for quality regardless of the level of readability and ability to be understood by the general population of Arabic speakers. The internet is a powerful yet two-edged tool when it comes to the health sector. Hence, governments, in collaboration with international and national health agencies/organizations, need to implement initiatives and take actions to ensure the dissemination of correct and reliable information on the internet. In order to achieve this, they have to support the visibility of reliable information to a greater extent, collaborate with scientific institutes or organizations with the aim of sharing reliable information, develop simple tools to assess the quality of information on websites, and use these assessments to discover and address misinformation and make it easier for users to find reliable information.

## Supplementary Information

**Additional file 1: Table S1** A list of eligible websites included for quality and readability analyses.

## Data Availability

The datasets supporting the findings of this article are available from the corresponding author.

## Abbreviations

COVID: Corona Virus Disease
HONcode: Health on the Net Foundation Code of Conduct
JAMA: The Journal of the American Medical Association
USDHHS: US Department of Health and Human Services
FKGL: Flesch Kincaid grade level
ARI: Automated Readability Index
SMOG: Simple Measure of Gobbledygook
FRE: Flesch Reading Ease
SD: Standard Deviation

## References

[1] WHO. Emergency Coronavirus disease (COVID-19) pandemic. Geneva: World Health Organization; 2020. Available at: https://www.who.int/home?gclid=EAIaIQobChMIteTTo%2D%2Dp7QIVWu3tCh0rsQ0AEAAYASAAEgL04PD_BwE (Accessed 20 November 2020).

[2] Sohrabi C, Alsafi Z, O'Neill N, Khan M, Kerwan A, Al-Jabir A (2020). World Health Organization declares global emergency: a review of the 2019 novel coronavirus (COVID-19). Int J Surg.

[3] Wong CKH, Wong JYH, Tang EHM, Au CH, Lau KTK, Wai AKC (2020). Impact of national containment measures on decelerating the increase in daily new cases of COVID-19 in 54 countries and 4 epicenters of the pandemic: comparative observational study. J Med Internet Res.

[4] Madurai Elavarasan R, Pugazhendhi R (2020). Restructured society and environment: a review on potential technological strategies to control the COVID-19 pandemic. SciTotal Environ.

[5] Effenberger M, Kronbichler A, Shin JI, Mayer G, Tilg H, Perco P (2020). Association of the COVID-19 pandemic with internet search volumes: a Google trends (TM) analysis. Int J Infect Dis.

[6] Li X, Liu Q (2020). Social media use, ehealth literacy, disease knowledge, and preventive behaviors in the COVID-19 pandemic: cross-sectional study on Chinese Netizens. J Med Internet Res.

[7] Craigie M, Loader B, Burrows R, Muncer S (2002). Reliability of health information on the **i**nternet: an examination of experts' ratings. J Med Internet Res.

[8] Cline RJ, Haynes KM (2001). Consumer health information seeking on the internet: the state of the art. Health Educ Res.

[9] Tan SS, Goonawardene N (2017). Internet health information seeking and the patient-physician relationship: a systematic review. J Med Internet Res.

[10] Cuan-Baltazar JY, Muñoz-Perez MJ, Robledo-Vega C, Pérez-Zepeda MF, Soto-Vega E (2020). Misinformation of COVID-19 on the internet: Infodemiology study. JMIR Public Health Surveill.

[11] Kulasegarah J, McGregor K, Mahadevan M (2018). Quality of information on the internet-has a decade made a difference?. Irish J Med Sci.

[12] Almaiman S, Bahkali S, Alabdulatif N, Bahkaly A, Al-Surimi K, Househ M (2016). Promoting oral health using social media platforms: seeking Arabic online oral health related information (OHRI). Stud Health Technol Inform..

[13] Alnemary FM, Alnemary FM, Alamri AS, Alamri YA. Characteristics of Arabic websites with information on autism. Neurosciences. (Riyadh) 2017; 22(2):143–145. 10.17712/nsj.2017.2.20160574 PMC5726822 28416788

[14] Alkhateeb JM, Alhadidi MS (2018). Information about epilepsy on the internet: an exploratory study of Arabic websites. Epilepsy Behav.

[15] Alnaim L (2019). Evaluation breast cancer information on the internet in Arabic. J Cancer Educ.

[16] Alakhali MS (2020). Quality assessment of information on oral cancer provided at Arabic speaking websites. Asian Pac J Cancer Prev.

[17] Charnock D, Shepperd S, Needham G, Gann R (1999). DISCERN: an instrument for judging the quality of written consumer health information on treatment choices. J Epidemiol Community Health.

[18] Silberg WM, Lundberg GD, Musacchio RA (1997). Assessing, controlling, and assuring the quality of medical information on the internet: Caveant lector et viewor--let the reader and viewer beware. JAMA..

[19] Boyer C, Baujard V, Geissbuhler A (2011). Evolution of health web certification through the HONcode experience. Stud Health Technol Inform.

[20] Edmunds MR, Barry RJ, Denniston AK (2013). Readability assessment of online ophthalmic patient information. JAMA Ophthalmol.

[21] Kher A, Johnson S, Griffith R (2017). Readability assessment of online patient education material on congestive heart failure. Adv Prev Med.

[22] Alhajj MN, Mashyakhy M, Ariffin Z, Ab-Ghani Z, Johari Y, Salim NS (2020). Quality and readability of web-based Arabic health information on denture hygiene: an infodemiology study. J Contemp Dent Pract.

[23] Borgmann H, Wölm JH, Vallo S, Mager R, Huber J, Breyer J (2017). Prostate cancer on the web-expedient tool for patients' decision-making?. J Cancer Educ.

[24] Sacchetti P, Zvara P, Plante MK (1999). The internet and patient education--resources and their reliability: focus on a select urologic topic. Urology..

[25] Nguyen SK, Ingledew PA (2013). Tangled in the breast cancer web: an evaluation of the usage of web-based information resources by breast cancer patients. J Cancer Educ.

[26] Janssen S, Fahlbusch FB, Käsmann L, Rades D, Vordermark D (2019). Radiotherapy for prostate cancer: DISCERN quality assessment of patient-oriented websites in 2018. BMC Urol.

[27] Case DO, Johnson JD, Andrews JE, Allard SL, Kelly KM (2004). From two-step flow to the internet: the changing array of sources for genetics information seeking. J Am Soc Inform Sci Technol..

[28] Stefl-Mabry J (2003). A social judgment analysis of information source preference profiles: an exploratory study to empirically represent media selection patterns. J Am Soc Inform Sci Technol.

[29] Jayasinghe R, Ranasinghe S, Jayarajah U, Seneviratne S (2020). Quality of online information for the general public on COVID-19. Patient Educ Couns.

[30] Weissenberger C, Jonassen S, Beranek-Chiu J, Neumann M, Müller D, Bartelt S (2004). Breast cancer: patient information needs reflected in English and German web sites. Br J Cancer.

[31] HON Code of Conduct for medical and health Web sites. Am J Health Syst Pharm. 2000;57(13):1283. 10.1093/ajhp/57.13.1283a. 10.1093/ajhp/57.13.1283a 10902071

[32] Hernández-García I, Giménez-Júlvez T (2020). Assessment of health information about COVID-19 prevention on the internet: Infodemiological study. JMIR Public Health Surveill.

